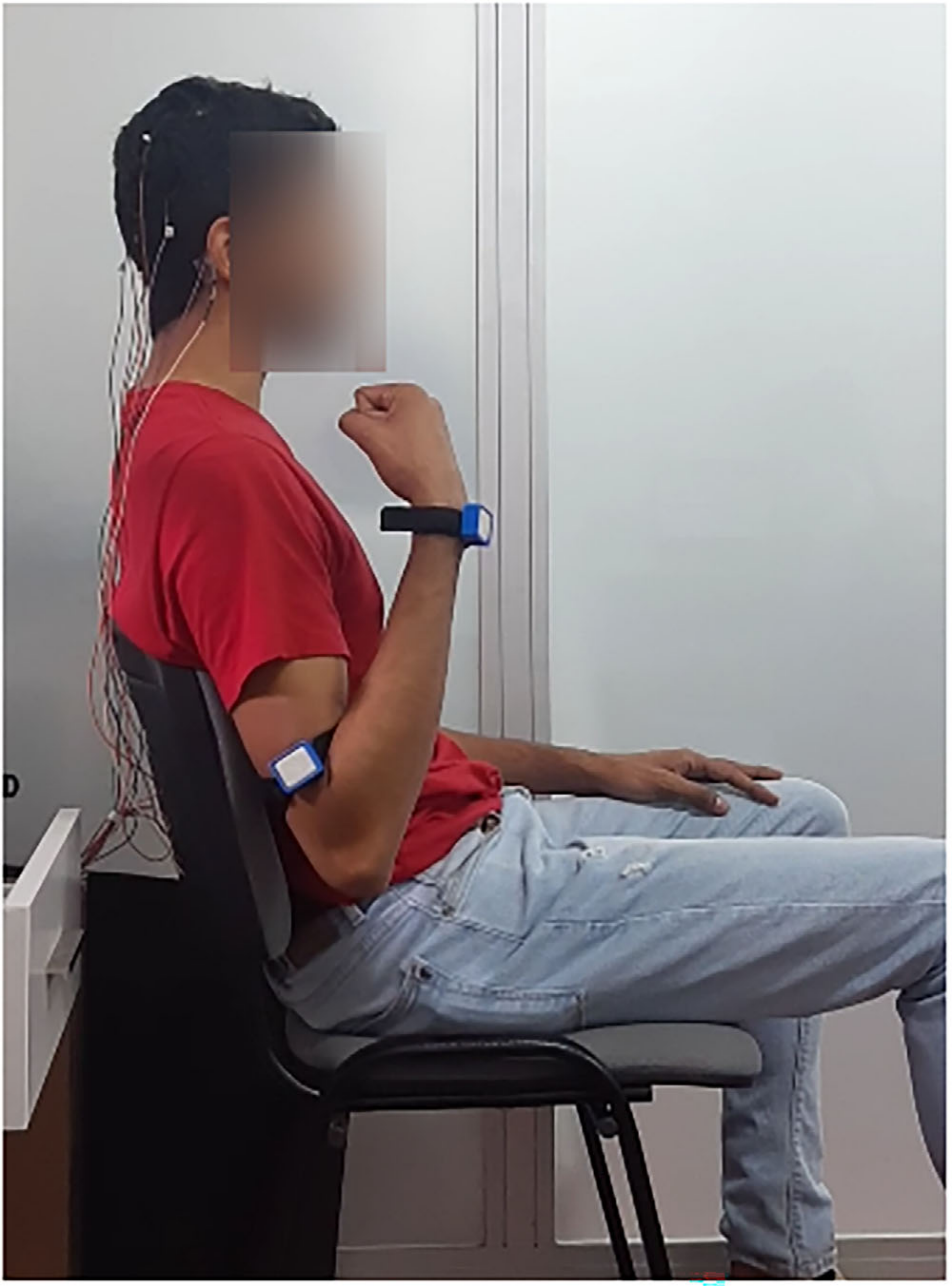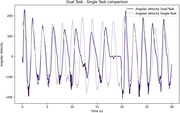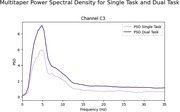# Implementation and validation of an electroencephalography dual‐task paradigm for detection of preclinical Alzheimer’s Disease

**DOI:** 10.1002/alz.095284

**Published:** 2025-01-09

**Authors:** Juliana Moreno Rada, Luisa María Zapata Saldarriaga, Eduardo Montoya Guevara, Santiago Gonzalez Cuartas, María José Hidalgo Ramírez, Paula Andrea Cataño Cano, Sara Garces Alvarez, Nicolás Vargas Flores, Juan Esteban Pineda Lopera, Santiago Rivera Estrada, Andrés Felipe Asprilla Mosquera, Juan Pablo Restrepo Mancilla, David Fernando Aguillón Niño, Juan Rafael Orozco Arroyave, Carlos Andrés Tobón Quintero, John Fredy Ochoa Gómez

**Affiliations:** ^1^ Universidad de Antioquia, Medellín, Antioquia Colombia; ^2^ Grupo Neuropsicología y Conducta GRUNECO, Medellín, Antioquia Colombia; ^3^ Semillero Neurociencias Computacionales NeuroCo, Medellín, Antioquia Colombia; ^4^ Grupo de Neurociencias de Antioquia GNA, Medellín, Antioquia Colombia; ^5^ Semillero de Investigación SINAPSIS, Medellín, Antioquia Colombia; ^6^ Grupo de Investigación en Telecomunicaciones Aplicadas GITA, Medellín, Antioquia Colombia

## Abstract

**Background:**

Dementia has a worldwide prevalence of 55 million people, with 60 to 70% of cases attributed to Alzheimer’s Disease (AD). In Antioquia, Colombia, exists a group of families with early‐onset AD associated to PSEN1‐E280A, a genetic variant with an autosomal dominant inheritance pattern and a penetrance over 99%, which enables the study of individuals across different disease stages. Electroencephalography (EEG) is a non‐invasive, portable, and low‐cost technique that allows the study of electrophysiological changes associated with neurodegeneration. When used in conjunction with dual‐task paradigms, EEG could serve as a clinical marker for evaluating brain functional reserve according to three theoretical models: the ability to share cognitive resources between tasks, the cross‐talk dependent on the content of processed information, and the bottleneck model. Therefore, the combined use of EEG and dual‐task represents a potential novel and adaptable alternative in clinical settings for detecting preclinical stages of AD.

**Method:**

The study population comprises cognitively healthy individuals who are carriers of the PSEN1‐E280A variant and control subjects without mobility limitations or impairments in verbal or written communication skills. The experimental protocol involves a motor task (single‐task) of elbow flexion‐extension and a motor‐cognitive task (dual‐task) that integrates the single‐task with six cognitive tasks. Data on upper extremity function (UEF) were collected using a tri‐axial wearable gyroscope and accelerometer sensor, while EEG recordings were obtained with the Open BCI CYTON BIOSENSING BOARD device, using the channels FP1, FP2, C3, C4, P7, P8, O1, O2.

**Result:**

10 healthy non‐carrier individuals have been evaluated to describe the behavior of the metrics in control subjects. The UEF measured by angular velocity curves illustrates a lower amplitude and flat points during the dual‐task compared to the single‐task, indicating that cognitive activity impacted motor performance. The EEG recordings showed a higher power spectral density during dual‐task compared to single‐task.

**Conclusion:**

The obtained results represent the motor performance between single and dual‐task modalities, which possibly represent a change in the dynamics of brain functioning as observed in the EEG signals.